# Importance of Ultraviolet-C (UV-C) Emitter Configuration for the Attenuation of Staphylococcus aureus and Candida auris Pathogens

**DOI:** 10.7759/cureus.71612

**Published:** 2024-10-16

**Authors:** Randy W Loftus, Carmen T Brindeiro, Franklin Dexter, Michelle C Parra, Soyun M Hwang, Brendan Wanta, Debra J Szeluga, Brent A Hadder, Melinda S Seering, Jonathan E Charnin

**Affiliations:** 1 Anesthesiology and Perioperative Medicine, Mayo Clinic, Rochester, USA; 2 Microbiology, RDB Bioinformatics, Coralville, USA; 3 Anesthesia, University of Iowa, Iowa City, USA

**Keywords:** candida auris, emitter configuration, pathogen attenuation, staphylococcus aureus, ultraviolet-c (uv-c), uv-c emitters

## Abstract

Background: The relative importance of different ultraviolet-C (UV-C) emitter configurations on the attenuation of vegetative bacterial and fungal pathogens has not been assessed. We hypothesized that emitter configuration would impact the efficacy of UV-C attenuation of *Staphylococcus aureus (S. aureus)* and *Candida auris* (*C. auris)* pathogens.

Methods: American Type Culture Collection (ATCC) *S.* *aureus *(ATCC 6538) and *C. auris* (ATCC MYA-5001*)* carriers (ReadyNowTM Test Carriers, Stratix Labs Corporation, Saint Paul, MN) were mounted on an aluminum stand along with three calibrated radiometers (International Light Technologies model ILT1270, Peabody, MA). Five UV-C emitter configurations were assessed, including three emitters with a triangular configuration about the stand and each rotating 360° (1), one emitter facing the stand and rotating 360° (2), three emitters facing the stand in a linear configuration and each rotating 5° (3), one emitter facing the stand and rotating 5° (4), and one emitter facing the stand and rotating 90° (5). Three serial experiments were conducted. The first experiment involved the establishment of the minimally effective irradiation dose (mean and standard deviation mJ/cm^2^) required to achieve no growth (6-log reduction (LR)) with direct exposure to pathogen carriers positioned at the center of the lamp. We then assessed the relative efficacy of delivery of the minimally effective dose via the five emitter configurations in attenuating polycarbonate and textured pathogen carriers. Polycarbonate carriers were positioned at 25.5 and 69.5 inches from the floor and oriented vertically to the emitters. Textured plastic pathogen carriers were positioned at 47.5 or 58.5 inches from the floor and with a 45° or horizontal orientation to the emitters. Standard carriers (1”x0.9”) were used for both pathogens and large carriers (1”x3”) for *C. auris*,the latter to address the potential for cell clustering.

Results: With standard carriers, the minimally effective dose was 27.01± 0.15 mJ/cm^2^ for *S. aureus* but was not achieved for *C. auris*. The minimally effective dose for large *C. auris* carriers was 596.62 ± 27.98 mJ/cm^2^. With standard carriers, all configurations achieved a >6 log reduction for *S. aureus,* and none achieved a >6 log reduction for *C. auris.* All configurations achieved a > 6 log reduction when 596.62 ± 27.98 mJ/cm^2^ was delivered to large *C. auris* carriers. Changing to textured plastic carriers (standard for *S. aureus* and large for *C. auris*) and varying height (47.5-69.5 inches) from the floor and orientation to the emitters (45° and horizontal), the mean ± standard deviation for *S. aureus* and *C. auris* log reductions with delivery of the minimally effective dose was 4.44 ± 2.02, 2.58 ± 2.37, 3.55 ± 2.67, 2.33 ± 2.47, and 3.00 ± 2.64 for configurations one through five, respectively. Configuration one achieved a significantly greater LR than configurations two (adjusted P = 0.0018) and four (adjusted P = 0.023). There were 22% (6/27) of sites ≥ 100 colony-forming units (CFU) following cleaning but before UV-C vs. 0% (0/27) of sites ≥ 100 CFU after surface disinfection cleaning and nine minutes of configuration three UV-C treatment (P = 0.023).

Conclusions: The choice of UV-C emitter configuration can impact *S. aureus* and *C. auris *attenuation when there is indirect exposure to the pathogen. Emitter configuration should be considered as an important parameter for future UV-C technological assessments.

## Introduction

While ultraviolet-C (UV-C) irradiation can reliably reduce environmental contamination [1, 2], the impact on healthcare-associated infections has been variable [3-6]. This could be explained in part by human factors (e.g., time limitations or use of only post-discharge cleaning) that inhibit the use of the technology [7]. However, recent work has shown that UV-C emitter positioning about the environmental target can impact efficacy [8]. An emitter configuration involving three towers positioned triangularly about a target, each rotating 360 degrees (configuration one), was shown to generate clinically relevant log reductions (LRs) in *Clostridioides difficile* (*C. difficile*) despite barriers to dose delivery (e.g., horizontal orientation to the emitters), while a more conventional configuration involving one emitter rotating 360 degrees (configuration two) was found to be the least reliable among the five different configurations tested [8]. Thus, the previously reported variability in UV-C efficacy for the prevention of healthcare-associated infections may be driven in part by the UV-C emitter configuration employed [3-8]. To further solidify this premise, the importance of UV-C configuration must be confirmed for other causative organisms for healthcare-associated infections. 

*Staphylococcus aureus* (*S. aureus*) and *Candida auris* (*C. auris*) transmission in the intensive care unit (ICU) environment [9] is associated with the development of bloodstream and ventilator-associated pneumonia (VAP) infections that increase patient mortality, hospital duration [10, 11], and healthcare expenditure [12]. A multifaceted approach to improved basic preventive measures is indicated, including enhanced environmental cleaning [13, 14]. Despite the solidified role of the environment as a potent transmission vehicle for these pathogens [15, 16], an evidence-based cleaning strategy for targeted attenuation of these pathogens has not been delineated.

Ultraviolet-C may be an effective strategy. Two recent studies [17, 18] assessed the efficacy of the triangular configuration one for attenuation of the more pathogenic *S. aureus* sequence type five (ST5) that is known to have increased strength of biofilm formation and desiccation tolerance [17]. In one study, the triangular configuration was shown to generate substantial reductions in ST5, requiring only a two-minute treatment period [17]. In another study, delivery of UV-C irradiation via the triangular configuration was found to be non-inferior to surface disinfection for attenuation of ST5 [18]. However, the relative efficacy of the UV-C emitter configuration has not been evaluated for either *S. aureus* or *C. auris* pathogens. Important configurations to consider are those that represent current technology. Two commonly employed configurations have been evaluated: a single emitter rotating 360 degrees [3-6] and an approach involving three emitters [17, 18]. Such information can help to guide future investigations into the efficacy of and/or the clinical application of UV-C technology for their prevention.

We hypothesized that like *C. difficile* [8], the triangular UV-C emitter configuration involving the use of three emitters would generate a greater log reduction for *S. aureus* and *C. auris *pathogens following delivery of a minimally effective dose under conditions of indirect irradiation exposure vs. a single emitter rotating 360 degrees. We aimed to assess the relative efficacy of five UV-C emitter configurations representative of current multi [17, 18] and single emitter technology [1] along with potential barriers to dose delivery [19] to test this hypothesis.

## Materials and methods

This study was conducted from April to August 2023. This experimental design did not include human or animal subjects, tissue, or samples, so it was exempt from approval of the local ethics committee, thus there was no number assigned. 

We anticipated attenuation of UV-C irradiation dose delivery due to planned assessment under conditions of indirect irradiation exposure (e.g., horizontal orientation to the emitters) in subsequent experiments [19]. As such, we first established a dose of UV-C irradiation that could reliably achieve a > 6-log reduction (no growth) from baseline controls for directly exposed pathogen carriers.

Commercially available polycarbonate carriers (ReadyNowTM Test Carriers, Stratix Labs Corporation, Saint Paul, MN) were obtained for *S. aureus* American Type Culture Collection (ATCC) 6538 and *C. auris* ATCC MYA-5001. This technology facilitates reproducible use of a standard test method (American Society for Testing and Materials (ASTM) 3135: Standard Practice for Determining Antimicrobial Efficacy of Ultraviolet Germicidal Irradiation Against Microorganisms on Carriers with Simulated Soil) for assessment of disinfection efficacy [20]. Per the manufacturer, the MB-06-10 ‘Standard Operating Procedure for Germicidal Spray Products as Disinfectants’ (GSPT) [21] and MB-25-05, ‘Standard Operating Procedure for OECD Quantitative Method for Evaluating Bactericidal and Mycobactericidal Activity of Microbicides Used on Hard, Non-Porous Surfaces’ [22]. Procedures were followed for the preparation of vegetative bacterial cells. Synthetic broth plus the addition of glucose was used where possible rather than nutrient broth or trypticase soy broth (MB-25-05) [22] due to the defined composition and improved consistency of synthetic broth as compared to undefined media such as nutrient broth and trypticase soy broth [22]. Growth conditions were executed according to EPA MB-06-10 [22] except for incubation time, which was followed according to MB-25-05 [22]. The 18-24-hour growth period as defined in MB-25-05 [22] was leveraged because it is expected to result in consistent growth of cells in the stationary phase rather than longer growth periods that may result in cells entering the death phase of the growth cycle. These procedures are considered to generate consistent preparation of viable microorganisms for antimicrobial testing, including for UV-C [21, 22]. Inoculum preparations for pathogen carriers were >8 log colony-forming unit (CFU)/mL, achieved by diluting or concentrating the cell suspension in the coating solution to ensure a >6 log CFU/carrier after plating and drying. The method for preparation of *C. auris* included growth on Sabouraud dextrose agar, Emmons (Remel, Lenexa, KS) for 48+/-4 hours at 35.0+/-1° C. The cells were then harvested from the agar to create an initial inoculum that was >8 log CFU/mL prior to carrier coating as described above by diluting or concentrating the cell suspension. Carriers were air dried until the droplets were no longer visible, for 15-20 minutes [20].

The top of the cartridge was removable to allow exposure of the pre-inoculated carrier to disinfection agents, and the back side of the cartridge had an adhesive strip for mounting the device to vertical test surfaces. These carriers were used for both treatment and control conditions, and all carriers were prepared, handled, and processed for CFU enumeration using identical procedures as outlined below.

The laboratory testing area was confirmed to be free from line-of-sight obstacles or obstructions. Three UV-C emitters using low-pressure mercury lamps (Surfacide, Waukesha, WI) were positioned in a row (linear configuration) facing an aluminum stand placed at nine feet (2.74 m) from the emitters. The distance to the center of the stand was measured via the use of a calibrated tape measure from the blue power inlet on the center of the middle emitter. The emitters were designed to provide coverage for the area of a typical patient bay [23].

**Figure 1:**
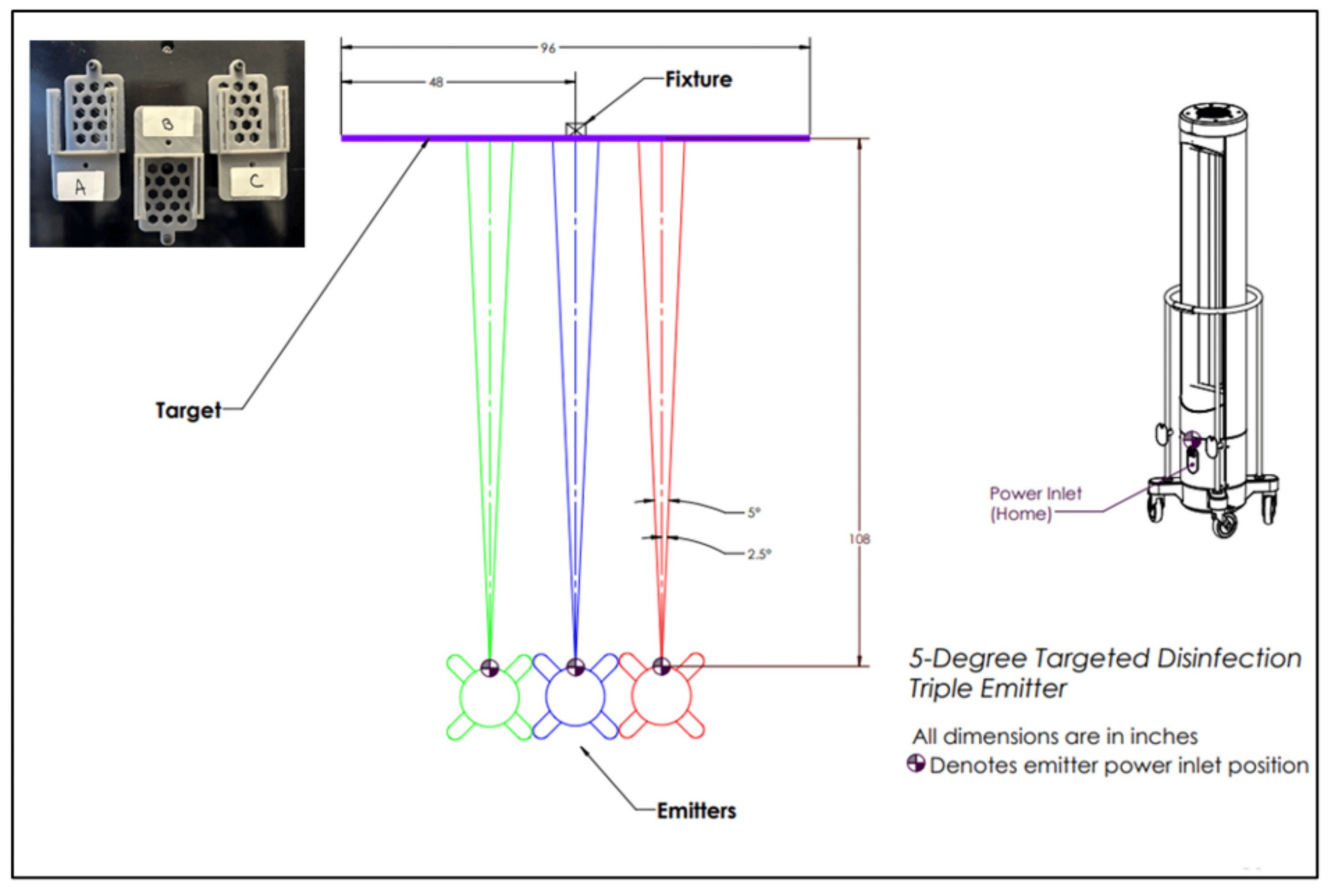
Ultraviolet-C (UV-C) dose response testing for American Type Culture Collection (ATCC) Staphylococcus aureus (ATCC 6538) and Candida auris (ATCC MYA-5001) Pathogen carriers were mounted on an aluminum stand (fixture) at three positions (A = center of stand, B = left of center of stand, C = right of center of stand) and at a height of 47.5 inches from the floor, center of bulbs. The carriers were positioned on the metal plate (upper left corner of the figure) that was attached to the stand via a screw (hole above position B) that allowed consistency of carrier positioning in relation to the center of the stand while adjusting the height and between experiments. The stand was positioned at nine feet from three low-pressure mercury gas UV-C emitters that were positioned in a row and each rotating five degrees. The test carriers and adjacent radiometers were exposed to an increasing dose of UV-C energy from 27.01 to 2018.17 (mJ/cm^2^). Log reductions (LR) were calculated by comparing the average of treatment samples (final colony-forming units (CFU)) for a given dose to the average of positive controls (initial CFU), log_10_(initial CFU/final CFU). Following treatment at each target dose, carrier and control slides were removed from the cartridge and processed identically to enumerate CFU. Log reductions were calculated by comparing the average of treatment samples (final CFU) for a given dose to the average of positive controls (initial CFU), log_10_(initial CFU/final CFU). This image was created by the authors.

The following process was applied to carriers of standard size (1”x 0.9”) for both *S. aureus* and *C. auris*. For each experiment, treatment pathogen carriers and previously calibrated radiometers (International Light Technologies model ILT1270, Peabody, MA) were mounted on the stand at positions A (left of center of stand), B (center of stand), and C (right of center of stand), facing and positioned directly ahead of the center emitter. The aluminum stand is denoted by a fixture (Figure 1).

Control pathogen carriers (N = 3 due to correlation with the three treatments) were opened for ambient environmental exposure and placed in a cardboard box that was covered by a tin pan (13 x 21 x 3 in) lined with aluminum foil and positioned immediately behind the emitters during the treatment period. Importantly, to measure the delivered radiation, the radiometers were always positioned vertically to the emitters, while the orientation of the carriers could be changed to assess efficacy. As the intensity of UV-C irradiation varies over the length of the bulbs, with the highest irradiance generated from the center of the bulbs, carriers, and radiometers were placed at the center of the bulbs, measured at 47.5 inches (120.65 cm) from the floor. The radiometers were calibrated to 254 nm irradiation and used to measure the band of wavelengths surrounding the peak intensity wavelength. Irradiance, W/cm^2^, or power/cm^2^, delivered to the carriers at a given distance was measured by the radiometers, where the dose delivered to the carriers was a function of time of irradiance exposure, W/cm^2^ X time (seconds) of exposure, or J/cm^2^. The UV-C emitters were turned on, allowed to warm up for 10 minutes outside of the test room, and returned to the marked locations. Three emitters positioned side-by-side, each rotating five degrees, were used to deliver energy to the carriers at the center of the lamp.

The test carriers and adjacent radiometers were exposed to an increasing dose of UV-C energy to establish a log reduction curve. Radiometers were connected, and the ILT software opened sequentially. For each radiometer, the display units were J/cm^2^. The emitters were turned off only after all three emitters had each reached each measured dose, with the average maximum cumulative dose for the three radiometers calculated and recorded.

The experiment above was repeated for *C. auris* with larger carriers (1”x3”) to address the potential of cell clustering. The inoculum and carrier surface concentration can impact germicidal performance data, in part related to cell clumping that occurs with inoculum above 10^6^ CFU/ml [24].

Following treatment at each target dose, the ATCC carrier polycarbonate slides, treatment, and control were removed from the cartridge and placed into a 50 mL conical tube (Thermo Fisher Scientific, Waltham, MA) containing 10 mL of phosphate-buffered saline (PBS) (Thermo Fisher Scientific), a 10^-3^ dilution. Conical tubes were vortexed at high speed for 30 seconds with the use of a timer, and serial dilutions were made as follows: 10 µl of solution to 990 µl of PBS and five seconds of vortexing on high speed, 10^-6^, and 100 µl of the 10^-6^ solution to 900 µl of PBS, 10^-7^. For aerobic gram-positive and gram-negative pathogens, each dilution (100 µl) was added to a 5% sheep’s blood agar (SBA) (Thermo Fisher Scientific) plate from the lowest to highest dilution. The SBA plates were then incubated at 36°C for 24 hours and CFUs quantified. For *C. auris*, the same process was followed, but plates were incubated at 40°C. Total CFU were counted at 48 hours, where ≥ 500 CFU were considered too numerous to count and recorded as 500. Log reductions were calculated by comparing the average of treatment samples in positions A, B, and C (final CFU) for each of the two dilutions (10^-6^ and 10^-7^ dilutions for each position, N = 6) for a given dose to the average of the three positive controls for each of the two dilutions (N = 6) (initial CFU), log_10_(initial CFU/final CFU). An effective dose was defined by the achievement of a > 6 log reduction for two consecutive doses.

To assess for photoreactivation and dark repair or excision of pyrimidine dimers caused by UV-C irradiation and bacterial regrowth [25], across the dose range, all *S. aureus* plates were exposed to visible light for three hours, incubated for an additional 24 hours in the incubator to allow for dark repair, and CFU recounted. There was no change in CFU (data not shown).

**Figure 2:**
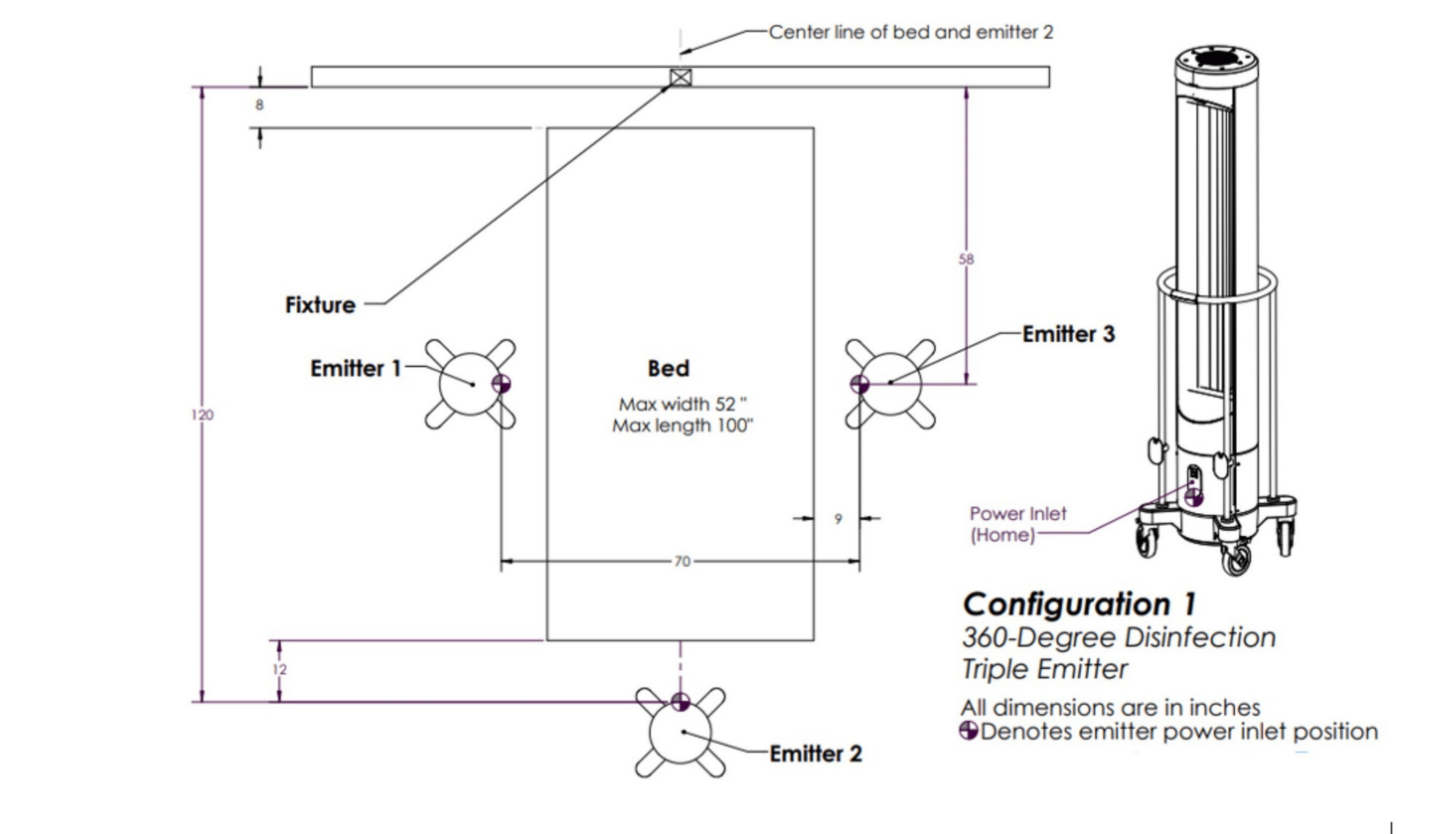
Ultraviolet-C (UV-C) emitter configuration one Three emitters are positioned triangularly about the target each and rotating 360 degrees. This image was created by the authors.

**Figure 3:**
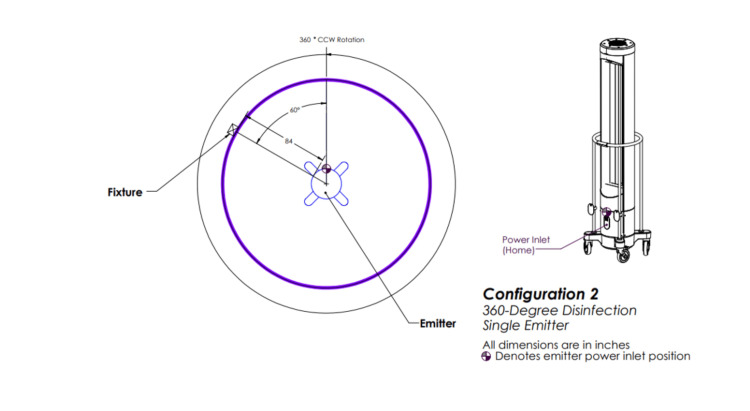
Ultraviolet-C (UV-C) emitter configuration two One emitter positioned in a room and rotating 360 degrees. This image was created by the authors.

**Figure 4:**
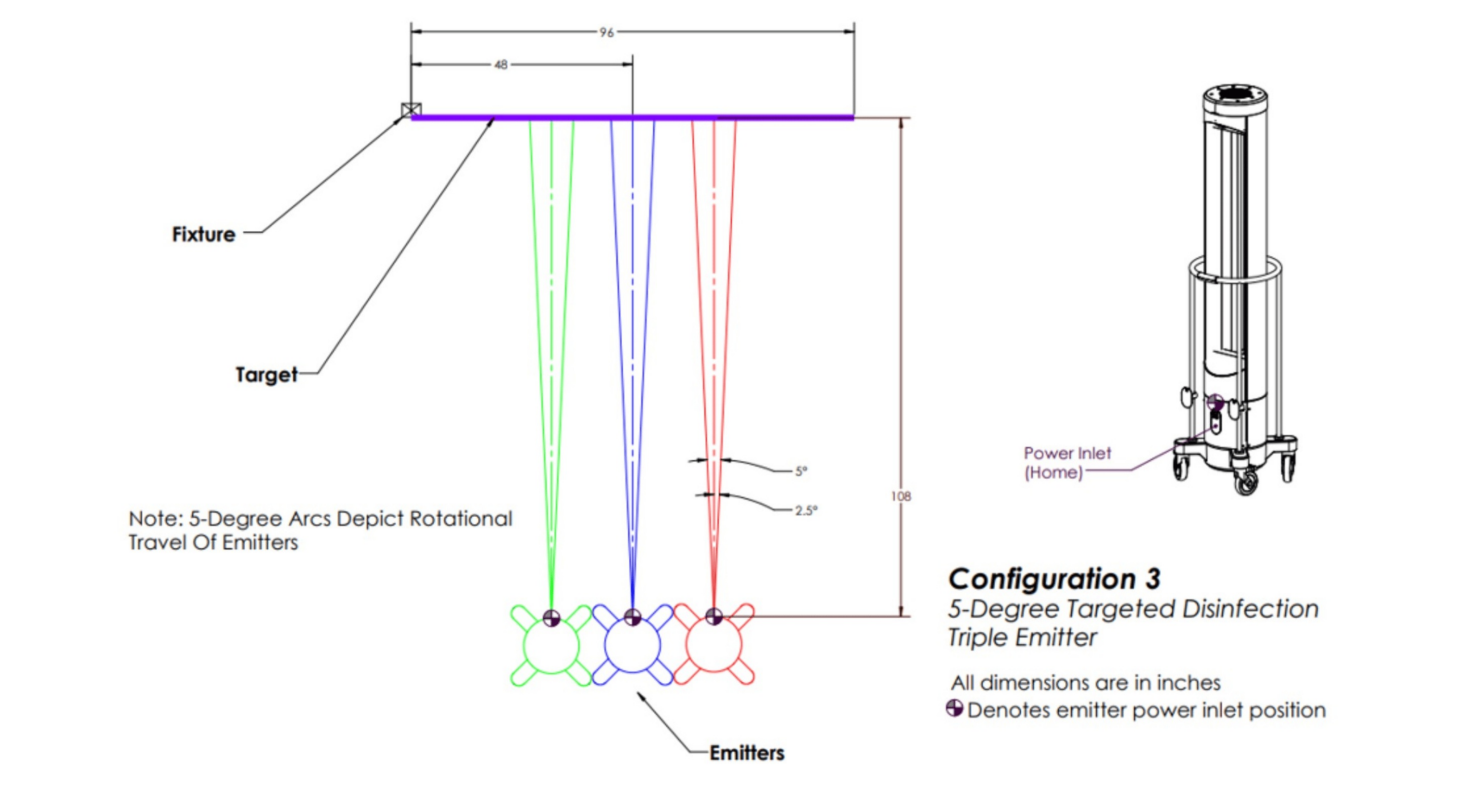
Ultraviolet-C (UV-C) emitter configuration three Three emitters are positioned in front of the target in a row each and rotating five degrees. This image was created by the authors.

**Figure 5:**
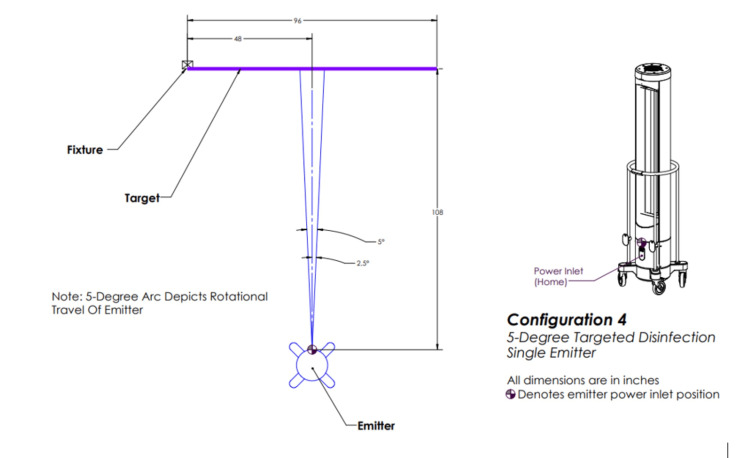
Ultraviolet-C (UV-C) emitter configuration four One emitter is positioned in front of the target and rotating five degrees. This image was created by the authors.

**Figure 6:**
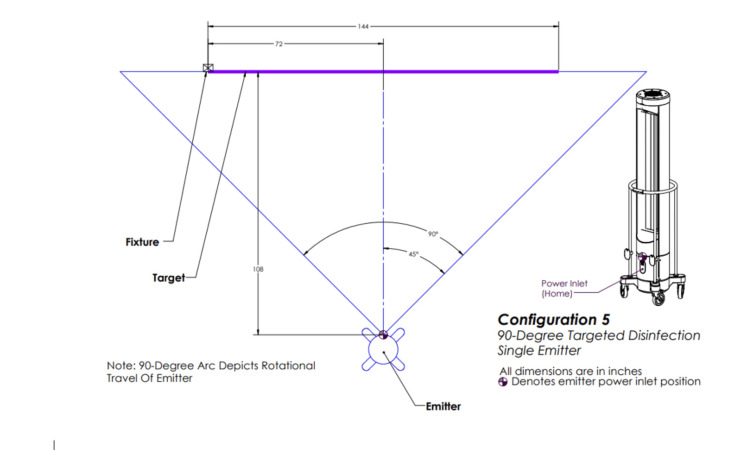
Ultraviolet-C (UV-C) emitter configuration five This image was created by the authors.

The above irradiation doses were then delivered to *S. aureus* and *C. auris* via each of the five configurations. Configuration one involved three emitters rotating 360 degrees (Figure 2), configuration two involved one emitter rotating 360 degrees (Figure 3), configuration three involved three emitters each rotating five degrees (Figure 4), configuration four involved one emitter rotating five degrees (Figure 5), and configuration five involved one emitter rotating 90 degrees (Figure 6).

Pathogen carriers oriented vertically to the emitters were positioned on a stand at nine feet from the emitters and at 25.5 and 69.5 inches from the floor. Soiling involved fetal bovine serum (FBS). Radiometers were positioned as above, and UV-C was turned off once the target dose for each pathogen was reached for the last of the three radiometers. The average maximum cumulative dose was recorded. Each carrier was immediately processed as described above, respective to the involved pathogen, and LR was calculated.

We then assessed via the approach above the potential impact of target height, orientation, and material [8,19] (polycarbonate or textured plastic, acrylonitrile butadiene styrene (ABS)) carriers (ReadyNow^TM^ Test Carriers, Stratix Labs Corporation) on attenuation of *S. aureus* and *C. auris*. Soiling remained FBS. We chose to examine 58.5 in from the floor under these conditions given that 69.5 cm, top of the lamp, with horizontal orientation, would be expected to generate zero attenuation, where 58.5 is midrange between 47.5 in (center of lamp) and 69.5 in (top of lamp). In this way, we could examine the impact of the angle of incidence on UV-C efficacy.

Using configuration three at nine feet and at 25.5, 47.5, and 58.5 inches from the floor, we simultaneously measured delivered irradiance (mJ/cm^2^) to radiometers oriented vertically and horizontally to the emitters until horizontal emitters reached 575 mJ/cm^2^.

The above data were analyzed to identify an evidence-based approach for the delivery of a sufficient dose for target pathogens in the ICU. We considered a typical ICU room to be approximately eight feet wide and nine feet deep with a central patient bed and monitors, and while facing the ICU room at the foot of the patient’s bed, an IV pump to the left of the patient and tables/equipment to the right of the patient [23]. We considered pathogens that would grow under aerobic conditions, including but not limited to *S. aureus* and *C. auris*, where we would expect delivery of approximately 575 mJ/cm^2^ to provide a broad range of coverage from the more susceptible *S. aureus* to the more resistant *C. auris*. We determined the cycle time required for dose delivery throughout the disinfection space (height and distance) for the applicable equipment configuration, lamp warm-up time (10 minutes), and emitter-to-emitter variation.

We identified three ICU rooms at the University of Iowa, Iowa City, that were at least eight feet wide and nine feet deep, had undergone terminal cleaning according to usual practices, and were ready for patient admission on August 8, 2023. Rooms included a private room in the surgical neurointensive care unit bay two, a shared room in the surgical neurosciences intensive care unit bay three, and a private room in the cardiovascular intensive care unit. Surface disinfection involved the use of a quaternary ammonium compound according to usual protocol, and the rooms were ready for patient occupancy. The UV-C treatment had to be such that patient admission would not be delayed.

Twenty-seven samples (nine for each of the three rooms) were obtained from a variety of surfaces and equipment materials at various heights and with various orientations to the emitters before and after UV-C treatment and within the eight-foot by nine-foot treatment area. These sample locations included the door handle, bedside tabletop, bedside table side, bedrail top, bedrail front, medication pump front, medication pump side, computer screen, and desk. Samples were collected using a dry ESwab (Copan, Murrieta, CA). If the sampled area was < 10x10 cm^2^, the entire surface area was sampled. If the sampled area was > 10 x 10 cm^2^, a 10 x 10 cm^2^ area was sampled. The samples were sent to the lab immediately, vortexed for five seconds on medium-high, 1:100 dilutions made, and 100 µl of each 1:100 dilution plated to SBA and incubated at 36°C (an appropriate temperature given that *C. auris* can grow well at up to 40°C) [26] for 24 hours. The CFUs were quantified for samples after surface disinfection but before UV-C and samples after surface disinfection and after UV-C.

Statistics

Average, maximum, and cumulative doses and associated LRs were calculated using simple descriptive statistics. Wilcoxon-Mann-Whitney tests were used to compare LRs for configuration one vs. each of the other four configurations. A Holm-Bonferroni adjustment was made for the four comparisons, treating adjusted P <0.05 as statistically significant. The proportion of measured sites returning ≥ 100 CFU after surface disinfection but before UV-C was compared via Fisher’s exact test to the proportion of the same measured sites returning ≥ 100 CFU [27, 28] after both surface disinfection and after nine minutes of treatment with UV-C via configuration three.

## Results

The minimal effective dose when using standard carriers was for *S. aureus* (27.01 ± 0.15 mJ/cm^2^). While a minimal effective dose was not achieved for *C. auris*, a > 6 log reduction was achieved once at approximately 2 J/cm^2^ (Table 1).

**Table 1:** Ultraviolet-C (UV-C) dose curve response by target pathogen Average dose refers to the average irradiation dose received for the three radiometers in positions A, B, and C. Positive control CFU is for the experiment, where there were three controls, one for each carrier position (A, B, C), and each position had two dilutions. Thus, the average was for six samples. Where control CFU is repeated, it was because the same control was used for the experiment. The same applies to the UV-C CFU/mL average. All carriers were placed nine feet from the emitters with vertical orientation and at 47.5 inches from the floor, the center of the lamp. Soiling was fetal bovine serum. Log reductions (LR) were calculated by comparing the average of treatment samples (final CFU) for a given dose to the average of positive controls (initial CFU), log_10 _(initial CFU/final CFU). Std = standard carrier; Lg = large carrier; SD = standard deviation; CFU = colony-forming unit

Organism	Average dose (mJ/cm^2^)	Dose SD	Positive control CFU/mL		LR
Staphylococcus aureus	27.01	0.15	3.07 x 10^8^	0	> 6
Staphylococcus aureus	53.6	0.73	3.07 x 10^8^	0	> 6
Staphylococcus aureus	78.56	0.83	3.07 x 10^8^	0	> 6
Staphylococcus aureus	103.88	1.08	3.07 x 10^8^	0	> 6
Staphylococcus aureus	129.83	0.94	3.07 x 10^8^	0	> 6
Staphylococcus aureus	154.65	0.47	3.07 x 10^8^	0	> 6
Staphylococcus aureus	178.74	1.29	3.07 x 10^8^	0	> 6
Staphylococcus aureus	204.07	1.57	3.07 x 10^8^	0	> 6
Staphylococcus aureus	230.16	1.49	3.07 x 10^8^	0	> 6
Staphylococcus aureus	253.14	1.87	3.07 x 10^8^	0	> 6
Candida auris	52.59	0.41	1.07 x 10^9^	4.98 x 10^8^	0.33
Candida auris	104.24	0.79	1.07 x 10^9^	3.58 x 10^8^	0.48
Candida auris	164.05	0.94	1.07 x 10^9^	2.33 x 10^8^	0.66
Candida auris	203.49	1.08	1.07 x 10^9^	2.68 x 10^8^	0.60
Candida auris	253.82	2.3	1.07 x 10^9^	1.92 x 10^8^	0.75
Candida auris	303.69	2.66	1.07 x 10^9^	1.58 x 10^8^	0.83
Candida auris	732.03	5.11	1.07 x 10^9^	3.82 x 107	1.45
Candida auris	1157.62	10.31	1.07 x 10^9^	2.17 x 10^7^	1.69
Candida auris	1584.54	13.04	1.07 x 10^9^	9.83 x 10^6^	2.04
Candida auris	2018.17	21.01	1.07 x 10^9^	0	> 6

With larger carriers, the minimal effective dose for *C. auris* was 596.62 ± 27.98 mJ/cm^2^ (Table 2).

**Table 2:** Impact of delivery of the minimally effective dose for large C. auris carriers Average dose refers to the average irradiation dose received for the three radiometers in positions A, B, and C. Positive control CFU is for the experiment, where there were three controls, one for each carrier position (A, B, C), and each position had two dilutions. Thus, the average was for six samples. The same applies to the UV-C CFU/mL average. All carriers were placed nine feet from the emitters with vertical orientation and at 47.5 inches from the floor, the center of the lamp. Soiling was fetal bovine serum. Log reductions (LR) were calculated by comparing the average of treatment samples (final CFU) for a given dose to the average of positive controls (initial CFU), log_10 _(initial CFU/final CFU). SD = standard deviation; CFU = colony-forming unit

Organism	Average dose (mJ/cm^2^)	Dose SD	Positive control CFU/mL	Average UV-C CFU/mL	LR
Candida auris	53.92	2.66	1.45 x 10^7^	0	>6
Candida auris	131.78	6.68	1.45 x 10^7^	1.67 x 10^5^	1.94
Candida auris	209.93	10.68	1.45 x 10^7^	0	>6
Candida auris	286.78	14.73	1.45 x 10^7^	0	>6
Candida auris	364.33	16.32	1.45 x 10^7^	0	>6
Candida auris	438.3	18.78	1.45 x 10^7^	3.33 x 10^5^	1.64
Candida auris	519.09	24.33	1.45 x 10^7^	1.67 x 10^5^	1.94
Candida auris	596.62	27.98	1.45 x 10^7^	0	>6
Candida auris	673.42	31.01	1.45 x 10^7^	0	>6
Candida auris	750.21	34.51	1.45 x 10^7^	0	>6

All configurations achieved a > 6 log reduction for *S. aureus* with the delivery of the minimally effective dose (26.58 ± 0.89 mJ/cm^2^) to standard, polycarbonate carriers positioned at nine feet from the emitters, at 25.5 and 69.5 in from the floor, and with vertical orientation to the emitters. There were no configurations that achieved a > 6 log reduction involving delivery of 2071.40 ± 33.83 mJ/cm^2^ to standard *C. auris* carriers (Table 3).

**Table 3:** Impact of UV-C emitter configuration on attenuation of polycarbonate pathogen carriers at nine feet and at 25.5 and 69.5 in from the floor Positive control (ctrl) is the average of three controls, one for each position A, B, and C, where each position had two dilutions, so for both control and UV-C treatment samples, N = six. Log reductions (LR) were calculated by comparing the average of treatment samples (final CFU) for a given dose to the average of positive controls (initial CFU), log_10 _(initial CFU/final CFU). For example, for *Candida auri*s configuration 1 UV-C 25.5 in, log10 (915,000,000/20,000,000) = log reduction of 1.66. #This is the average LR for the two heights of 25.5 in and 69.5 in from the floor. std: standard; mJ/cm2 = average irradiance for the 3 radiometers, positions A, B, and C; ctrl = control; UV-C = ultraviolet-C; LR = log reduction; CFU = colony-forming unit

Configuration	Organism std carriers	25.5 in mJ/cm^2^	69.5 in mJ/cm^2^	Pos Ctrl CFU/mL	UV-C 25.5 in CFU/mL	LR	UV-C 69.5 in CFU/mL	LR	LR^#^
One	Staphylococcus aureus	28.38	26.35	6.67 x 10^7^	0	> 6	0	>6	>6
One	Candida auris	2101	2087	9.15 x 10^8^	2.00 x 10^7^	1.66	2.05 x 10^7^	1.65	1.66
Two	Staphylococcus aureus	27.54	25.16	1.38 x 10^8^	0	>6	0	>6	>6
Two	Candida auris	2075.7	2032.8	1.13 x 10^9^	2.27 x 10^7^	1.70	3.83 x 10^6^	2.47	2.09
Three	Staphylococcus aureus	26.22	26.64	1.22 x 10^8^	0	>6	0	>6	>6
Three	Candida auris	2040.31	2025.45	1.08 x 10^9^	8.50 x 10^6^	2.10	1.53 x 10^7^	1.85	1.98
Four	Staphylococcus aureus	26.91	26.6	9.23 x 10^7^	0	>6	0	>6	>6
Four	Candida auris	2118.17	2110.83	1.40 x 10^9^	2.52 x 10^7^	1.74	1.35 x 10^7^	2.02	1.88
Five	Staphylococcus aureus	26.13	25.89	1.84 x 10^8^	0	>6	0	>6	>6
Five	Candida auris	2079.47	2043.3	1.07 x 10^9^	8.33 x 10^6^	2.11	2.18 x 10^7^	1.69	1.90

All configurations achieved a > 6 log reduction for *C. auris* when 606.18 ± 9.59 mJ/cm^2^ were delivered to large polycarbonate carriers positioned at nine feet from the emitters, at 25.5 and 69.5 in from the floor, and with vertical orientation to the emitters (Table 4).

**Table 4:** Impact of UV-C emitter configuration on attenuation of large Candida auris carriers Lg = large; Pos = positive; Ht = height; mJ/cm^2^ is the average irradiance for each of the three carrier positions, including A (left of center), B (center), and C (right of center); Ctrl = control; CFU = colony-forming unit; Pos Ctrl CFU is the mean CFU for each of the three carrier controls, where if the number is the same, it is because there was the same control for the given experiment; LR = log reduction

Configuration	Organism	Ht (in)	mJ/cm^2^	Pos Ctrl CFU	UV-C CFU	LR
One	Candida auris	25.5	606.45	9.83 x 10^6^	0	>6
One	Candida auris	69.5	600.03	9.83 x 10^6^	0	>6
Two	Candida auris	25.5	597.55	3.65 x 10^7^	0	>6
Two	Candida auris	69.5	592.08	3.65 x 10^7^	0	>6
Three	Candida auris	25.5	618.85	9.62 x 10^7^	0	>6
Three	Candida auris	69.5	615.75	9.62 x 10^7^	0	>6
Four	Candida auris	25.5	620.37	3.03 x 10^7^	0	>6
Four	Candida auris	69.5	608.87	3.03 x 10^7^	0	>6
Five	Candida auris	25.5	600.17	9.83 x 10^6^	0	>6
Five	Candida auris	69.5	601.66	9.83 x 10^6^	0	>6

When the carrier substrate was changed to textured plastic, height varied from 47.5 to 58.5 in, and orientation to the emitters varied from 45 degrees to horizontal, the mean effective doses (mJ/cm^2^) were comparable among configurations one to five: 311 ± 286 (N = 52), 304 ± 282 (N = 24), 322 ± 316 (N = 8), 316 ± 308 (N = 8), and 311 ± 304 (N = 8), respectively. The log_10_ reduction and standard deviation achieved with delivery of 27.63 ± 1.56 mJ/cm^2^ to standard size *S. aureus* carriers and 594.05 ± 11.82 mJ/cm^2^ to large *C. auris *carriers (the LRs generated under a variety of conditions were averaged for each pathogen by configuration) were 4.44 ± 2.02, 2.58 ± 2.37, 3.55 ± 2.67, 2.33 ± 2.47, and 3.00 ± 2.64 for configurations one through five, respectively (Table 5).

**Table 5:** Impact of UV-C configuration on pathogen attenuation when carrier substrate was changed to textured plastic, height varied from 47.5 to 58.5 inches, and orientation to the emitters varied from 45 degrees to horizonal Position = radiometers positioned at different spots in the nine-foot circumference of the rotating emitters; Orient = orientation, how the pathogen carriers were oriented to the ultraviolet-C (UV-C) emitters; Height = Ht (inches) is the distance from the floor for the pathogen carriers when placed on the aluminum stand; UV-C dose is the mean for the maximum cumulative dose delivered to each of the three radiometers in positions A (left of center of stand), B (center of stand), and C (right of center of stand); SD = standard deviation, Ctrl = control carriers, and CFU = colony-forming units; LR = log reduction. Ctrl CFU is the mean CFU for each of the three controls for the experiment, where the Ctrl is repeated. It is so because those were the controls for the same experiment.

Configuration	Organism	Position	Orient	Ht (in)	UV-C dose	SD Dose	Ctrl CFU	SD Ctrl CFU	UV-C CFU	SD UV-C CFU	LR
One	Candida auris	1	Horizontal	58.5	585.75	10.38	4.58 x 10^7^	1.7 x 10^7^	1.67 x 10^5^	4.08 x 10^5^	2.43
One	Candida auris	1	45 degrees	58.5	588.71	13.94	4.58 x 10^7^	1.7 x 10^7^	0	N/A	6
One	Candida auris	1	45 degrees	47.5	597.83	13.56	4.58 x 10^7^	1.7 x 10^7^	0	N/A	6
One	Candida auris	1	Horizontal	47.5	587.09	10.35	4.58 x 10^7^	1.7 x 10^7^	0	N/A	6
One	Candida auris	2	45 degrees	47.5	588.67	8.3	4.58 x 10^7^	1.7 x 10^7^	0	N/A	6
One	Candida auris	2	Horizontal	47.5	589.68	6.68	4.58 x 10^7^	1.7 x 10^7^	0	N/A	6
One	Candida auris	2	45 degrees	58.5	588.85	8.67	4.58 x 10^7^	1.7 x 10^7^	0	N/A	6
One	Candida auris	2	Horizontal	58.5	587.96	11.47	4.58 x 10^7^	1.7 x 10^7^	0	N/A	6
One	Candida auris	3	Horizontal	58.5	598.88	20.56	4.58 x 10^7^	1.39 x 10^7^	1.67 x 10^5^	4.08 x 10^5^	2.44
One	Candida auris	3	45 degrees	58.5	601.64	26.22	4.58 x 10^7^	13,909,230	0	N/A	6
One	Candida auris	3	45 degrees	47.5	593.59	16.82	4.58 x 10^7^	13,909,230	0	N/A	6
One	Candida auris	3	Horizontal	47.5	589.45	12.48	4.58 x 10^7^	13,909,230	0	N/A	6
One	Candida auris	4	Horizontal	47.5	598.57	20.7	4.58 x 10^7^	13,909,230	0	N/A	6
One	Candida auris	4	45 degrees	47.5	615.32	32.15	4.58 x 10^7^	13,909,230	0	N/A	6
One	Candida auris	4	45 degrees	58.5	606.21	26.74	4.58 x 10^7^	13,909,230	0	N/A	6
One	Candida auris	4	Horizontal	58.5	605.44	27.32	4.58 x 10^7^	1.39 x 10^7^	1.67 x 10^5^	4.08 x 10^5^	2.44
One	Candida auris	5	Horizontal	58.5	598.6	19.25	4.58 x 10^7^	1.39 x 10^7^	0	N/A	6
One	Candida auris	5	45 degrees	58.5	602.41	21.9	4.58 x 10^7^	1.39 x 10^7^	0	N/A	6
One	Candida auris	5	45 degrees	47.5	610.79	30.08	4.58 x 10^7^	1.39 x 10^7^	0	N/A	6
One	Candida auris	5	Horizontal	47.5	600.59	23.57	4.58 x 10^7^	1.39 x 10^7^	1.67 x 10^5^	4.08 x 10^5^	2.44
One	Candida auris	6	Horizontal	47.5	586.55	10.46	3.65 x 10^7^	3.65 x 10^7^	0	N/A	6
One	Candida auris	6	45 degrees	47.5	584.42	16.46	3.65 x 10^7^	3.65 x 10^7^	0	N/A	6
One	Candida auris	6	45 degrees	58.5	590.66	16.34	3.65 x 10^7^	3.65 x 10^7^	0	N/A	6
One	Candida auris	6	Horizontal	58.5	587.32	12.98	3.65 x 10^7^	3.65 x 10^7^	5.00 x 10^5^	1.22 x 10^6^	1.86
One	Candida auris	7	Horizontal	47.5	588.63	12.69	3.65 x 10^7^	3.65 x 10^7^	0	N/A	6
One	Candida auris	7	45 degrees	47.5	593.25	16.11	3.65 x 10^7^	3.65 x 10^7^	0	N/A	6
One	Staphylococcus aureus	1	45 degrees	47.5	26.75	0.49	1.39 x 10^8^	6.49 x 10^7^	1.67 x 10^5^	4.08 x 10^5^	2.92
One	Staphylococcus aureus	1	Horizontal	47.5	26.16	0.32	1.39 x 10^8^	6.49 x 10^7^	6.2 x 10^6^	4.49 x 10^6^	1.35
One	Staphylococcus aureus	1	Horizontal	58.5	26.57	0.35	1.39 x 10^8^	6.49 x 10^7^	1.32 x 10^7^	1.32 x 107	1.02
One	Staphylococcus aureus	1	45 degrees	58.5	27.1	0.64	1.39 x 10^8^	6.49 x 10^7^	0	N/A	6
One	Staphylococcus aureus	2	45 degrees	58.5	29.84	3	1.39 x 10^8^	6.49 x 10^7^	0	N/A	6
One	Staphylococcus aureus	2	Horizontal	58.5	29.75	3.35	1.39 x 10^8^	6.49 x 10^7^	4.83 x 10^6^	4.22 x 10^6^	1.46
One	Staphylococcus aureus	2	Horizontal	47.5	29.54	2.84	1.39 x 10^8^	6.49 x 10^7^	3.0 x 10^6^	3.90 x 10^6^	1.67
One	Staphylococcus aureus	2	45 degrees	47.5	28.2	2.38	1.39 x 10^8^	6.49 x 10^7^	0	N/A	6
One	Staphylococcus aureus	3	45 degrees	47.5	25.05	0.49	1.39 x 10^8^	6.49 x 10^7^	0	N/A	6
One	Staphylococcus aureus	3	Horizontal	47.5	25.54	0.3	1.39 x 10^8^	6.49 x 10^7^	6.83 x 10^6^	7.65 x 10^6^	1.31
One	Staphylococcus aureus	3	Horizontal	58.5	25.51	0.39	1.39 x 10^8^	6.49 x 10^7^	6.17 x 10^6^	5.31 x 10^6^	1.35
One	Staphylococcus aureus	3	45 degrees	58.5	26.67	0.51	1.39 x 10^8^	6.49 x 10^7^	0	N/A	6
One	Staphylococcus aureus	4	45 Degrees	58.5	28.29	1.93	1.26 x 10^8^	5.67 x 10^7^	1.67 x 10^5^	4.08 x 10^5^	2.88
One	Staphylococcus aureus	4	Horizontal	58.5	29.39	1.82	1.26 x 10^8^	5.67 x 10^7^	7.00 x 10^6^	7.56 x 10^6^	1.26
One	Staphylococcus aureus	4	Horizontal	47.5	28.21	1.76	1.26 x 10^8^	5.67 x 10^7^	1.83 x 10^6^	2.86 x 10^6^	1.84
One	Staphylococcus aureus	4	45 degrees	47.5	32.13	2.55	1.26 x 10^8^	5.67 x 10^7^	0	N/A	6
One	Staphylococcus aureus	5	45 degrees	47.5	27.61	0.98	1.26 x 10^8^	5.67 x 10^7^	0	N/A	6
One	Staphylococcus aureus	5	Horizontal	47.5	28.5	1.37	1.26 x 10^8^	5.67 x 10^7^	1.33 x 10^6^	2.42 x 10^6^	1.98
One	Staphylococcus aureus	5	Horizontal	58.5	31.42	1.8	1.26 x 10^8^	5.67 x 10^7^	2.83 x 10^6^	3.25 x 10^6^	1.65
One	Staphylococcus aureus	5	45 degrees	58.5	29.17	1.64	1.26 x 10^8^	5.67 x 10^7^	1.67 x 10^5^	4.08 x 10^5^	2.88
One	Staphylococcus aureus	6	45 degrees	58.5	29.15	1.18	1.26 x 10^8^	5.67 x 10^7^	0	N/A	6
One	Staphylococcus aureus	6	Horizontal	58.5	29.75	2.09	1.26 x 10^8^	5.67 x 10^7^	3.17 x 10^6^	5.04 x 10^6^	1.6
One	Staphylococcus aureus	6	Horizontal	47.5	27.85	1.35	1.26 x 10^8^	5.67 x 10^7^	5.00 x 10^5^	8.37 x 10^5^	2.4
One	Staphylococcus aureus	6	45 degrees	47.5	26.99	1.35	1.26 x 10^8^	5.67 x 10^7^	0	N/A	6
One	Staphylococcus aureus	7	45 degrees	47.5	29.59	1.36	1.26 x 10^8^	5.67 x 10^7^	0	N/A	6
One	Staphylococcus aureus	7	Horizontal	47.5	28.38	0.93	1.26 x 10^8^	5.67 x 10^7^	0	N/A	6
Two	Candida auris	N/A	Horizontal	58.5	579.43	4.11	1.5 x 10^7^	9.63 x 10^6^	7.17 x 10^6^	9.09 x 10^6^	0.32
Two	Candida auris	N/A	Horizontal	25.5	584.33	6.3	1.5 x 10^7^	9.63 x 10^6^	5.0 x 10^5^	5.48 x 10^5^	1.48
Two	Candida auris	N/A	Horizontal	47.5	578.64	2.73	1.5 x 10^7^	9.63 x 10^6^	2.83 x 10^6^	3.82 x 10^6^	0.72
Two	Candida auris	N/A	Horizontal	58.5	585.06	8.91	1.5 x 10^7^	9.63 x 10^6^	1.10 x 10^7^	9.7 x 10^7^	0.13
Two	Candida auris	N/A	45 degrees	47.5	579.91	4.24	3.72 x 10^7^	2.10 x 10^8^	0	N/A	6
Two	Candida auris	N/A	45 degrees	58.5	577.18	2.94	3.72 x 10^7^	2.10 x 10^8^	0	N/A	6
Two	Candida auris	N/A	Horizontal	47.5	580.1	6.5	2.68 x 10^7^	1.52 x 10^7^	1.67 x 10^5^	4.08 x 10^5^	2.21
Two	Candida auris	N/A	Horizontal	58.5	579.55	4.1	2.68 x 10^7^	1.52 x 10^7^	4.17 x 10^6^	3.76 x 10^6^	0.81
Two	Candida auris	N/A	45 degrees	47.5	579.75	3.73	1.25 x 10^7^	9.16 x 10^6^	0	N/A	6
Two	Candida auris	N/A	45 degrees	58.5	578.62	2.32	1.25 x 10^7^	9.16 x 10^6^	0	N/A	6
Two	Candida auris	N/A	Horizontal	58.5	580.38	3.28	1.25 x 10^7^	9.16 x 10^6^	0	N/A	6
Two	Candida auris	N/A	Horizontal	47.5	583.79	7.22	1.25 x 10^7^	9.16 x 10^6^	1.67 x 10^6^	4.08 x 10^6^	0.88
Two	Staphylococcus aureus	N/A	45 degrees	47.5	29.96	0.73	7.8 x 10^7^	4.54 x 10^7^	0	N/A	6
Two	Staphylococcus aureus	N/A	45 degrees	58.5	26.85	0.78	7.8 x 10^7^	4.54 x 10^7^	3.33 x 10^5^	8.16 x 10^5^	2.37
Two	Staphylococcus aureus	N/A	Horizontal	58.5	29.58	0.82	2.99 x 10^8^	1.61 x 10^8^	1.41 x 10^8^	5.27 x 10^7^	0.33
Two	Staphylococcus aureus	N/A	Horizontal	47.5	26.8	0.49	2.99 x 10^8^	1.61 x 10^8^	6.75 x 10^7^	2.71 x 10^7^	0.65
Two	Staphylococcus aureus	N/A	Horizontal	25.5	27.22	0.72	1.67 x 10^8^	9.62 x 10^7^	4.33 x 10^6^	5.09 x 10^7^	1.59
Two	Staphylococcus aureus	N/A	Horizontal	58.5	26.53	0.88	1.67 x 10^8^	9.62 x 10^7^	1.72 x 10^8^	1.33 x 10^7^	0
Two	Staphylococcus aureus	N/A	Horizontal	58.5	27.01	0.53	1.67 x 10^8^	9.62 x 10^7^	6.30 x 10^7^	2.37 x 10^7^	0.42
Two	Staphylococcus aureus	N/A	Horizontal	47.5	26.89	0.59	1.67 x 10^8^	9.62 x 10^7^	2.75 x 10^7^	2.05 x 10^7^	0.78
Two	Staphylococcus aureus	N/A	Horizontal	58.5	26.45	0.75	3.47 x 10^8^	1.19 x 10^8^	3.67 x 10^6^	4.27 x 10^6^	1.98
Two	Staphylococcus aureus	N/A	Horizontal	47.5	25.38	0.27	3.47 x 10^8^	1.19 x 10^8^	3.33 x 10^6^	4.89 x 10^6^	2.02
Two	Staphylococcus aureus	N/A	45 degrees	58.5	26.44	0.55	3.47 x 10^8^	1.19 x 10^8^	0	N/A	6
Two	Staphylococcus aureus	N/A	45 degrees	47.5	26.51	0.79	3.47 x 10^8^	1.19 x 10^8^	1.67 x 10^5^	4.08 x 10^5^	3.32
Three	Candida auris	N/A	45 degrees	47.5	613.26	36.6	3.15 x 10^8^	1.80 x 10^7^	0	N/A	6
Three	Candida auris	N/A	45 degrees	58.5	616.42	37.06	3.15 x 10^8^	1.80 x 10^7^	0	N/A	6
Three	Candida auris	N/A	Horizontal	47.5	616.65	38.99	3.15 x 10^8^	1.80 x 10^7^	1.67 x 10^5^	4.08 x 10^5^	2.28
Three	Candida auris	N/A	Horizontal	58.5	625.83	46.69	3.15 x 10^8^	1.80 x 10^7^	3.12 x 10^6^	4.02 x 10^6^	2.00
Three	Staphylococcus aureus	N/A	45 Degrees	47.5	27.09	1.34	2.53 x 10^8^	1.29 x 10^8^	0	N/A	6
Three	Staphylococcus aureus	N/A	45 Degrees	58.5	26.98	1.14	2.53 x 10^8^	1.29 x 10^8^	0	N/A	6
Three	Staphylococcus aureus	N/A	Horizontal	47.5	26.58	0.86	2.53 x 10^8^	1.29 x 10^8^	5.42 x 10^7^	3.07 x 10^7^	0.67
Three	Staphylococcus aureus	N/A	Horizontal	58.5	27.25	1.3	2.53 x 10^8^	1.29 x 10^8^	8.80 x 10^7^	2.11 x 10^7^	0.46
Four	Candida auris	N/A	Horizontal	58.5	604.26	25.29	1.58 x 10^8^	8.30 x 10^7^	4.17 x 10^6^	4.62 x 10^6^	1.58
Four	Candida auris	N/A	45 Degrees	58.5	604.59	25.5	1.58 x 10^8^	8.30 x 10^7^	0	N/A	6
Four	Candida auris	N/A	45 Degrees	47.5	605.63	26.42	1.58 x 10^8^	8.30 x 10^7^	3.98 x 10^7^	1.32 x 10^7^	0.60
Four	Candida auris	N/A	Horizontal	47.5	602.01	23.61	1.58 x 10^8^	8.30 x 10^7^	1.0 x 10^6^	1.26 x 10^6^	2.2
Four	Staphylococcus aureus	N/A	45 Degrees	47.5	27.82	2.47	2.34 x 10^8^	1.49 x 10^8^	0	N/A	6
Four	Staphylococcus aureus	N/A	45 Degrees	58.5	27.6	2.41	2.34 x 10^8^	1.49 x 10^8^	1.67 x 10^5^	4.08 x 10^5^	3.15
Four	Staphylococcus aureus	N/A	Horizontal	47.5	27.27	1.91	2.34 x 10^8^	1.49 x 10^8^	1.42 x 10^7^	1.39 x 10^7^	1.22
Four	Staphylococcus aureus	N/A	Horizontal	58.5	27.41	1.83	2.34 x 10^8^	1.49 x 10^8^	2.97 x 10^7^	1.10 x 10^7^	0.9
Five	Candida auris	N/A	45 Degrees	58.5	596.99	20.41	1.72 x 10^7^	1.01 x 10^7^	0	N/A	6
Five	Candida auris	N/A	45 Degrees	47.5	596.2	21.1	1.72 x 10^7^	1.01 x 10^7^	0	N/A	6
Five	Candida auris	N/A	Horizontal	47.5	591.94	15.22	1.72 x 10^7^	1.01 x 10^7^	3.33 x 10^5^	5.12 x 10^5^	1.71
Five	Candida auris	N/A	Horizontal	58.5	595.22	18.28	1.72 x 10^7^	1.01 x 10^7^	3.5 x 10^6^	3.94 x 10^6^	0.69
Five	Staphylococcus aureus	N/A	45 Degrees	58.5	26.21	2.71	3.77 x 10^7^	1.64 x 10^8^	3.33 x 10^5^	8.17 x 10^5^	2.05
Five	Staphylococcus aureus	N/A	Horizontal	47.5	25.91	0.49	1.38 x 10^8^	1.58 x 10^7^	5.9 x 10^7^	4.22 x 10^7^	0.37
Five	Staphylococcus aureus	N/A	Horizontal	58.5	26.54	0.44	1.38 x 10^8^	1.58 x 10^7^	8.4 x 10^7^	5.78 x 10^7^	0.22
Five	Staphylococcus aureus	N/A	45 Degrees	47.5	26.22	1.18	7.80 x 10^7^	4.54 x 10^7^	0	N/A	6

Configuration one had the highest observed LR, which was significantly greater than configurations two (adjusted P = 0.0018) and four (adjusted P = 0.023), but not configurations three (adjusted P = 0.22) or five (P = 0.19).

Treatment times for delivery of 575 mJ/cm^2^ for configurations one through five, respectively, were 48, 84, 12, 32, and 59 minutes. When approximately 575 mJ/cm^2^ was delivered via configuration three to radiometers positioned at nine feet, at 25.5, 47.5, and 58.5 inches from the floor, and with horizontal orientation to the emitters, the mean dose that was simultaneously delivered to radiometers with vertical orientation to the emitters across the three heights from the floor was 27.82 J/cm2 (Table 6).

**Table 6:** Configuration 3 delivery of irradiation (MJ/cm2) to targets with vertical and horizontal orientation Configuration three: three emitters in a row, each with 5° rotation; target dose: 575 mJ/cm^2^ delivered to a space 8 feet wide at nine feet

Radiometer	Target dose	Dose horizontal radiometer	Dose vertical radiometer
25.5” horizontal	575 mJ/cm^2^	575 mJ/cm^2^	6.27 J/cm^2^
47.5” horizontal	575 mJ/cm^2^	575 mJ/cm^2^	24.67 J/cm^2^
58.5” horizontal	575 mJ/cm^2^	575 mJ/cm^2^	52.58 J/cm^2^

There were 22% (6/27) of frequently touched ICU sites that returned ≥ 100 CFU following cleaning but before UV-C treatment and 0% (0/27) of sites that returned ≥ 100 CFU after surface disinfection cleaning followed by nine minutes of UV-C treatment with configuration three, two-sided P = 0.023 (Table 7).

**Table 7:** Practical assessment of clinical implementation of configuration three Fisher’s exact test was performed using the Stata v18.5 command “csi 6 0 21 27, exact” (StataCorp, College Station, TX); “6” and “0” are the CFU > 0 in the “PRE” and the “POST” categories, respectively; “21” and “27” are the CFU = 0 in the two categories, respectively. The two-sided P = 0.023. CFU: colony-forming units

Bay	Reservoir	CFU
1	Door handle PRE	0
1	Bedside tabletop PRE	500
1	Bedside table front PRE	200
1	Bedrail top PRE	1,200
1	Bedrail front PRE	0
1	Med pump front PRE	0
1	Med pump side PRE	0
1	Computer screen PRE	0
1	Computer desk PRE	0
1	Door handle POST	0
1	Bedside tabletop POST	0
1	Bedside table front POST	0
1	Bedrail top POST	0
1	Bedrail front POST	0
1	Med pump front POST	0
1	Med pump side POST	0
1	Computer screen POST	0
1	Computer desk POST	0
2	Sink handle PRE	100
2	Bedside tabletop PRE	0
2	Bedside table front PRE	200
2	Monitor handle PRE	0
2	Monitor screen PRE	0
2	Vent screen PRE	0
2	Vent handle PRE	0
2	Computer screen PRE	0
2	Computer desk PRE	0
2	Sink handle POST	0
2	Bedside tabletop POST	0
2	Bedside table front POST	0
2	Monitor handle POST	0
2	Monitor screen POST	0
2	Vent screen POST	0
2	Vent handle POST	0
2	Computer screen POST	0
2	Computer desk POST	0
3	Door handle PRE	0
3	Bedside tabletop PRE	0
3	Bedside table front PRE	0
3	Bedrail top PRE	0
3	Bedrail front PRE	0
3	Med pump front PRE	0
3	Med pump side PRE	100
3	Computer screen PRE	0
3	Computer desk PRE	0
3	Door handle POST	0
3	Bedside tabletop POST	0
3	Bedside table front POST	0
3	Bedrail top POST	0
3	Bedrail front POST	0
3	Med pump front POST	0
3	Med pump side POST	0
3	Computer screen POST	0
3	Computer desk POST	0

Eighteen distinct isolates were recovered, including two gram-negative rods, five gram-positive rods, and 11 gram-positive cocci.

## Discussion

The development of an evidence-based UV-C implementation strategy is indicated to address variability in the efficacy of UV-C for the prevention of healthcare-associated infections [3-6, 8]. Recent work has shown that UV-C emitter configuration is an important parameter that can impact efficacy for *C. difficile *attenuation in the laboratory setting [8]. A triangular configuration of three emitters positioned about the environmental target and rotating 360 degrees (configuration one) was shown to achieve a higher log reduction than the more conventional approach involving a single emitter rotating 360 degrees (configuration two) that demonstrated the lowest log reduction [8]. Based on this prior work, we tested the hypothesis that UV-C emitter configuration would also impact the attenuation of other causative organisms of infection for healthcare-associated infections to further solidify earlier results [8]. In this study, we examined the relative efficacy of five emitter configurations that were studied previously [8] in attenuating *S. aureus* and *C. auris* pathogens in a laboratory setting. We found again that configuration one achieved the highest log reduction and configuration two achieved the lowest LR. These study results support the conclusion of earlier work [8] that UV-C emitter configuration is an important consideration for future study design and/or practical implementation of UV-C technology.

Recent work assessed the relative efficacy of five UV-C emitter configurations for attenuation of *C. difficile*, an anaerobic, spore-forming pathogen with intrinsic UV-C resistance [8]. One limitation of this prior work was the focus on a single pathogen class. We extend prior work in this study by examining the impact of emitter configuration on aerobic, vegetative *(S. aureus*) and aerobic, fungal (*C. auris*) pathogens that have clinical relevance regarding the development of ventilator-associated pneumonia and central line-associated bloodstream infections [9-13]. Using *C. auris*, we examined the potential impact that pathogen clumping may have on UV-C irradiation efficacy, and we assessed the parallel delivery of horizontal and vertical irradiation during the delivery of a target dose. We then utilized the above findings to determine what would be an evidence-based and practical deployment strategy to assess UV-C augmentation of surface disinfection for frequently touched sites in the ICU environment.

For both* S. aureus *and *C. auris*, we found that, like prior work [8], the impact of emitter configuration can be delineated in the setting of barriers to dose delivery. For *S. aureus*, all configurations achieved a > 6 log reduction regardless of changes in height from the floor when a minimally effective dose was delivered to standard-size polycarbonate carriers with vertical orientation to the emitters. For *C. auris*, we found that all configurations failed to achieve a 6 log reduction as defined in this study with a delivery of up to approximately 2 J/cm^2^ to standard-sized carriers. When we adjusted to larger carriers for *C. auris* given the consideration of cell clustering [24], a 6 log reduction was consistently observed for all configurations. When the change was made to textured plastic carriers with variation in carrier orientation to the emitters, we found that configuration one achieved the highest mean LR, which was significantly greater than configurations two and four but no different than configurations three and five. Configuration two had the lowest LR. Thus, these study results validate earlier results [8] and support the primary hypothesis of this study that UV-C emitter configuration one has the potential to generate greater pathogen attenuation than conventional configuration two under conditions that limit irradiation exposure. We now know that this concept extends beyond the anaerobic *C. difficile* pathogen to aerobic vegetative and fungal pathogens. In addition, these findings support the concept that pathogen clustering is a potential barrier to UV-C efficacy [24], but they also suggest it is potentially modifiable. We considered increasing the plate size as a parallel to “debulking” with surface disinfection cleaning. Finally, we showed again [8] that when a target dose is delivered to a horizontal surface, in this case approximately 575 mJ/cm^2^, a substantially larger dose is delivered to vertical surfaces. In this case, the delivered vertical dose ranged from approximately 6,000 mJ/cm^2^ to 53 J/cm^2^ depending on height from the floor. Not only do these findings suggest that there is a risk of overtreatment and potentially associated photoreactivation and dark repair that may lead to subsequent regrowth [25], but there is also the potential for targeted attenuation of such a downstream effect. In the latter case, targeted surface disinfection following UV-C could be applied to objects at greater heights. Thus, from this work arises a novel concept, not only may UV-C augment surface disinfection, but there may be potential for surface disinfection to augment UV-C. Further work is indicated.

Taken together, UV-C implementation requires a scientific approach to the delivery of the technology. Prior work suggested that evidence-based UV-C implementation strategies should consider pathogen burden, intended log reduction, treatment area, other barriers to dose delivery (e.g., orientation to the emitters), and human factors (e.g., treatment time) in determining the UV-C configuration employed [8]. Regarding log reduction, we found in this study that while configuration one generated a higher log kill than configurations two and four, it was statistically no different than configurations three and five. Thus, configurations two and four were initially ruled out, leaving configurations one, three, and five. Prior work suggested that for frequently touched sites in the ICU, at least a 0.7-log reduction was indicated for anaerobic pathogens to reduce 500 CFU below 99 CFU. While this would reduce contamination below a clinically relevant threshold for surface hygiene [8], it was stated that a three-log reduction, 500 CFU to 1 CFU (essentially no growth), would be more desirable [8]. In a similarly fast-paced environment, the operating room, recent work showed that residual contamination could be as high as 10,000 CFU, where reducing 10,000 to 1 CFU would require a 4 log reduction [18]. Based on our study results, configurations one, three, and five could achieve log reductions within this range of 0.7 to 4 log despite barriers, but configuration three required the shortest treatment time of 12 minutes. The treatment time for configuration three for *C. difficile* was previously reported to require 8 minutes at a lower dose requirement to achieve a > 6 log reduction with direct irradiation, approximately 432 mJ/cm^2 ^[8]. Configuration five required a treatment time of 59 minutes and was limited in the treatment area. Thus, we selected configuration three as the evidence-based approach for configuration deployment to treat a typical ICU bay.

Using configuration three involving three UV-C emitters placed in a row in front of the ICU room, each emitter rotating five degrees, all target objects were within the range [23], and we felt that barriers such as horizontal orientation, height, etc. were sufficiently accounted for. We found that the eight minutes of treatment time was indeed important, as room turnover would not have allowed additional time per nursing staff, as, during the study, a patient was literally on the way to the ICU room. We show that the approach was effective in augmenting surface disinfection, generating at least a 4 log reduction based on the results. As the sites with residual contamination included horizontal surfaces, yet a substantial reduction was achieved, the results are in direct alignment with the study findings, including that there was no apparent effect of clustering that we could detect. We surmise that this relates to prior surface disinfection, highlighting the importance. Alternatively, it could be that pathogen clustering is unlikely on larger environmental surfaces in the clinical environment and only applies to laboratory testing. Future work should examine the potential for regrowth [25] with configuration three vs. configuration one, the impact of surface disinfection augmentation of UV-C, and whether clustering is a true clinical phenomenon.

The main limitation of this study is that it involves a laboratory assessment. The parallel strength of the laboratory approach is that it validates prior findings [8] and provides further impetus for the implementation and assessment of an evidence-based UV-C implementation strategy as part of a multifaceted program to generate reliable healthcare-associated infections reduction [8]. An additional limitation is that while we tested configurations that represent clinically available options, we did not test all conceivable configurations or equipment. However, the aim of this study was to validate the premise that the approach to UV-C implementation, specifically emitter configuration, can impact the efficacy of pathogen attenuation in a laboratory setting. The results of this study are however generalizable as long as the irradiation dose delivered by a variety of devices is monitored and confirmed to achieve at a distance of nine feet the minimally effective dose as described in this study. Realizing that dose delivery may not be monitored and that it can vary by device, future studies can leverage the methodology employed in this study to assess the impact of configuration for alternative technologies and/or assess different configurations (e.g., ceiling-based and/or automated robotic movement) in the laboratory and assess the clinical impact. Furthermore, future work may include ICU rooms that vary not only in size but also in the number of medical monitors and equipment in the room. We did not test all pathogens, but we studied previously anaerobic, spore-forming pathogens of clinical relevance [8], and we added to the prior work in this study by extending the analysis to aerobic vegetative and fungal pathogens. These findings were not necessarily expected, as it could have been that pathogen sensitivity to UV-C may have prevented the ability to detect differences in configuration. In fact, we saw that indeed, with direct exposure, all configurations performed at a similar level. It was only with barriers to dose delivery that we could decipher a difference. This conceptual framework can be applied to earlier work [3-6]. If one only evaluates the efficacy of UV-C at a short distance (e.g., less than two meters), it may be that factors such as dose delivery to indirectly exposed surfaces and/or overtreatment of directly exposed surfaces are lost. These findings should provide impetus for more careful considerations regarding future technological implementation. We recognize that we tested only three ICU rooms. The goal was not to show that there is a definitive augmentation of cleaning with UV-C but to show that logic can be applied to configuration choice to achieve a desired outcome. Future work is indicated to assess the clinical impact of this conceptual framework on *C. difficile*, ventilator-associated pneumonia, and central line-associated bloodstream infections. While residual pathogens following clinical application were not speciated, the point of the approach was to reduce overall microbial burden based on clinically relevant reductions [27, 28]. One might argue that if there were no detection of major bacterial pathogens, the cleaning was effective, but this would be a poor argument given that only 20% of pathogens are collected on sampled surfaces [29]. A more logical argument was that zero following UV-C was not truly zero, an argument that can be addressed statistically with a larger sample size [30]. However, this is a moot point for this study, as there were a variety of pathogens detected, some of which were gram-negative and including *Enterococcus*. Furthermore, if it were to be the case that in this study surface disinfection alone eradicated all detectable pathogens, that premise would not be supported by evidence showing repeatedly that surface disinfection fails [27].

## Conclusions

With the delivery of a minimally effective irradiation dose to *S. aureus* and *C. auris* pathogen carriers positioned at a distance of nine feet from and at various heights and orientations to UV-C emitters, UV-C configuration one (three emitters positioned triangularly about the stand and each rotating 360 degrees) achieved the highest observed log reduction for both pathogens. The log reduction achieved by configuration one was significantly greater than configurations two (one emitter facing the stand and rotating 360 degrees) and four (one emitter facing the stand rotating five degrees) but was not statistically different than that achieved by configurations three (three emitters positioned in a row and facing the stand) and five (one emitter facing the stand and rotating 90 degrees). Configuration three required the shortest time for delivery of the minimally effective irradiation dose among the five configurations. Nine minutes of treatment with configuration three for augmentation of usual intensive care unit cleaning were observed to be practical and efficacious. These findings support careful consideration of emitter configuration for future UV-C clinical applications and/or studies.
